# Assessing equity of access and affordability of care among South Sudanese refugees and host communities in two districts in Uganda: a cross-sectional survey

**DOI:** 10.1186/s12913-022-08547-5

**Published:** 2022-09-16

**Authors:** Jessica King, Pallavi Prabhakar, Neha Singh, Munshi Sulaiman, Giulia Greco, Sandra Mounier-Jack, Josephine Borghi

**Affiliations:** 1 London School of Hygiene and Tropical Medicine, London, UK; 2 Norwegian School of Economics, Bergen, Norway; 3 BRAC Uganda, Kampala, Uganda

**Keywords:** Refugees, catastrophic expenditure, health equity, maternal health

## Abstract

**Background:**

The vast majority of refugees are hosted in low and middle income countries (LMICs), which are already struggling to finance and achieve universal health coverage for their own populations. While there is mounting evidence of barriers to health care access facing refugees, there is more limited evidence on equity in access to and affordability of care across refugee and host populations. The objective of this study was to examine equity in terms of health needs, service utilisation, and health care payments both within and between South Sudanese refugees and hosts communities (Ugandan nationals), in two districts of Uganda.

**Methods:**

Participants were recruited from host and refugee villages from Arua and Kiryandongo districts. Twenty host villages and 20 refugee villages were randomly selected from each district, and 30 households were sampled from each village, with a target sample size of 2400 households. The survey measured condition incidence, health care seeking and health care expenditure outcomes related to acute and chronic illness and maternal care. Equity was assessed descriptively in relation to household consumption expenditure quintiles, and using concentration indices and Kakwani indices (for expenditure outcomes). We also measured the incidence of catastrophic health expenditure- payments for healthcare and impoverishment effects of expenditure across wealth quintiles.

**Results:**

There was higher health need for acute and chronic conditions in wealthier groups, while maternal care need was greater among poorer groups for refugees and hosts. Service coverage for acute, chronic and antenatal care was similar among hosts and refugee communities. However, lower levels of delivery care access for hosts remain. Although maternal care services are now largely affordable in Uganda among the studied communities, and service access is generally pro-poor, the costs of acute and chronic care can be substantial and regressive and are largely responsible for catastrophic expenditures, with service access benefiting wealthier groups.

**Conclusions:**

Efforts are needed to enhance access among the poorest for acute and chronic care and reduce associated out-of-pocket payments and their impoverishing effects. Further research examining cost drivers and potential financing arrangements to offset these will be important.

## Background

The right to health for refugees is a topic of major importance in global health: in 2021, there were 26.6 million refugees and an estimated 84 million forcibly displaced people worldwide [1]. Physical and mental healthcare needs for refugees can be substantial [2–4], and climate change is expected to increase the number of refugees to between 140 and 200 million by 2050 [5]. The vast majority (85%) of refugees are hosted in low and middle income countries (LMICs) [6], which are already struggling to finance and achieve universal health coverage (UHC) for their own populations [7]. There is substantial variation in health financing arrangements for refugees. In some countries international agencies finance refugee health care through cash transfers or vouchers [8], in others, refugees can access government funded free care [9], with services often being provided by non-governmental organisations (NGOs) or other non-public facilities receiving external financial support. As a result, the humanitarian response in host LMICs can often lead to ‘two-tier’ health systems for refugees and hosts, creating inequity in access to and affordability of healthcare [10], highlighting the need to consider the effect of financing arrangements for refugees on host communities as well as refugees themselves.

While there is mounting evidence of health care access barriers facing refugees, there is more limited evidence comparing access to healthcare among refugees and host communities. Some studies have found that refugee exclusion from health insurance schemes results in higher unmet need and reduced health care access compared to host populations [11], and that refugees face higher costs of care [12–14]. Other studies report refugees receiving inferior quality of care as a result of discrimination [9]. However, studies of access to maternal and reproductive health services in Uganda [15] and globally [16] have found that refugees have better access and lower maternal and neonatal mortality compared to host communities, or that levels of access to health care are quite similar [17].

Measuring affordability or financial protection is a key part of monitoring equitable access to health and progress towards UHC, and provides information on the financial hardship that may be caused by using health services [18]. One widely used measure of financial protection is the incidence of catastrophic health expenditure, or payments for healthcare which are higher than the resources of the household [19]. Catastrophic expenditure can be the result of outpatient or inpatient care [20], as well as maternal care seeking [21]. An alternative measure is the incidence of impoverishment due to out-of-pocket health payments, which measures the extent to which households are pushed below the poverty line because of their health expenditure [18].

To our knowledge, there have been very few studies systematically analysing affordability of care across refugee and host populations [22]. Existing work in Uganda either does not include refugees [23], or only examines access [24], or cost [25], but not affordability. Yet a lack of insurance coverage is more likely among poorer groups [26], and hence, access constraints may be unevenly distributed across wealth groups. Furthermore, despite the removal of user fees in Ugandan public health centres and hospitals in 2001, out-of-pocket health expenditure has remained high [27].

The objective of this study was to examine equity in terms of health needs, service utilisation, and health care payments both within and between South Sudanese refugees and host communities (Ugandan nationals), in two districts of northwest Uganda.

## Methods

### Study setting

Uganda is host to the third largest population of refugees globally, and the largest in sub-Saharan Africa, estimated at 1.5 million people [1]. Approximately 960,000 of these originate from South Sudan [28], displaced as a result of ongoing violence which broke out in December 2013 [29]. The vast majority of South Sudanese refugees live in seven settlements alongside host communities in the northwest of the country [30].

Uganda has a pluralistic health system, with government health facilities (which provide care without formal user fees) operating alongside private and not-for-profit organisations [27]. In 2018, the year of the latest available national health accounts, out of pocket payments constituted 41% of total health expenditure, while government expenditure represented just 16% [31]. Historically, refugees had access to dedicated primary healthcare facilities which were managed and funded by the United Nations High Commissioner for Refugees (UNHCR), with access to government hospital referral services. This resulted in perceived inequities between host and refugee communities in service access and tensions between these communities. However, from the early 2000s, refugee and host health services were integrated into a single health system under local government control. All health facilities in refugee settlements are now owned by the Ugandan government, and health services are run and funded by the government, with additional financial and material support provided by the UNHCR and other humanitarian actors towards health services within refugee districts [10, 32, 33]. The integration of refugee and host health services means that host populations can access the same health facilities as refugees for free [34, 35].

South Sudanese refugees in Uganda have substantial need for mental health and psychosocial support as a result of their experience of conflict and displacement [36]. There is also evidence that refugees in Uganda have greater health needs than those in host communities, with 51 percent of refugee households in Uganda defined as in need, compared to 17 percent of host households [37].

This study was conducted in two districts in Uganda: Arua, in the Northern region of the country, and Kiryandongo, in the Western region. Both districts are home to sizeable settlements of South Sudanese refugees: Rhino Camp (in Arua) has a population of 131,000, and the Kiryandongo refugee settlement has a population of 75,000 [28] . Rhino Camp is 70km from the main host town Arua, while the Kiryandongo settlement is just 5km from Bweyale town. Refugees in Rhino Camp tend to be more recent arrivals than those in Kiryandongo, who have generally been living in Uganda longer [28].

### Sampling

Participants were recruited from host and refugee villages from Arua and Kiryandongo districts. Every village was defined as either a host village if more than 50% of the population were Ugandan nationals, or a refugee village otherwise. A two-stage sampling approach was taken. Twenty host villages and 20 refugee villages were randomly selected from each district. Thirty households from each village were selected randomly through door-to-door household visits, and were eligible for inclusion if the household contained at least one woman of reproductive age (15-49 years). In host villages, only host households were eligible for inclusion in the survey, and in refugee villages only refugee households were eligible. In households with more than one woman of reproductive age, a pre-assigned table of random numbers was used to randomly select an interviewee, so no two women were interviewed from the same household [38]. A sample size calculation was undertaken based on estimating a prevalence of 50% for any given outcome, 30 respondents per cluster and intra-cluster correlation coefficient of 0.1 (design effect 3.9), and a 95% confidence interval and margin of error of 4%. This gave a target sample size of 1200 refugee and 1200 host women of reproductive age, or 80 villages.

### Data collection

The survey was conducted through in-person interviews in English and Arabic, and data was entered on tablets using SurveyCTO platform. Interviews lasted approximately 45 minutes and were conducted by a team of 25 trained enumerators in July-August 2019. Data were collected on individual and household characteristics, healthcare need, care seeking and costs of care as outlined below.

### Outcomes

We examined equity across illness incidence as a measure of health need, health care seeking and health care expenditure outcomes.

We included three measures of healthcare need, for acute sickness, chronic sickness and maternal care. Households were defined as having experienced acute sickness if anyone in the household had a short-term illness in the two weeks preceding the survey, and chronic sickness if anyone in the household had a long-lasting illness in the preceding month. Acute sickness was defined as an illness or injury that occurs suddenly with a rapid onset, and tends to resolve quickly on their own or with medical treatment, or is so severe and fast acting that a patient does not survive. Chronic sickness was defined as an illness which has a slow progression that builds over time, and tends to be a long lasting problem requiring multiple visits to a health facility. Women were defined as having need for maternal care if they reported having delivered a baby in Uganda in the preceding year.

Care seeking for households with acute or chronic sickness was defined as the sick household member having attended a public health centre, hospital or private clinic (excluding traditional healers, herbalists and community health workers) during the recall period (two weeks for acute sickness and one month for chronic sickness) .

The two measures of cares seeking for maternal care were four or more antenatal care (ANC) visits, and facility-based delivery. Women were defined as having had four or more ANC visits if they reported seeking ANC at least four times during their pregnancy, regardless of timing or facility type. They were defined as having a facility-based delivery if the baby was delivered at a health centre, hospital or private clinic.

Annual healthcare expenditures were estimated for acute and chronic care. Annual acute and chronic care costs were defined as the total expenditure on medicines, tests and consultation fees for any acute or chronic sickness in the household in the preceding two weeks (for acute care) or one month (chronic care), multiplied by 26 (acute care) or 12 (chronic care) to estimate annual expenditures. Maternal care costs were defined as the total expenditure on all reported components of ANC received before a delivery in Uganda in the last year and all costs of the delivery (including fees, and medicines).

We estimated equity in relation to household wealth, measured as reported monthly household consumption expenditure, and adjusted per adult equivalent. Consumption expenditure was considered a more reliable measure of ability to pay than income, which is often under-reported in developing countries [39].

We generated two measures of financial protection to monitor progress towards UHC, catastrophic health expenditure and impoverishment by health expenditure. Catastrophic expenditure was defined for categories of care (acute, chronic, and maternal) and all care at two thresholds, 10% and 25% of household expenditure.

Households were defined as poor if their daily consumption expenditure per adult equivalent was below the international poverty line of 1.9USD per day (2011 PPP prices). The number of adult equivalents in the household was calculated using the formula *adult equivalents* = (*children* × 0.33 + *adults*)^0.9^. As household size was reported categorically, households reporting 5 or 6 members were given a nominal size of 5.5, and households reporting 9+ members a nominal size of 9. As we did not have numbers of children and adults in the household separately, the number of household members who were adults was estimated indirectly from other questions about the respondent’s circumstances. We did this by summing the respondent herself, and any adults the respondent reported living with either as husbands, non-husband heads of household, or other women of reproductive age, as the number of adults. All other members of the household were assumed to be children. Households were defined as being impoverished by health expenditure if their adult equivalent consumption expenditure exceeded $1.90 per day, but subtracting their total annualised health expenditure from their total annualised expenditure pushed them below the international poverty line.

We generated quintiles of household consumption expenditure by ranking households based on expenditure per adult equivalent and dividing them into five equally sized groups from richest to poorest.

### Data analysis

We first described the characteristics of the overall sample, as well as separately for refugees and hosts, using descriptive statistics. We compared these to the same statistics drawn from the 2016 Demographic and Health Survey for the national Ugandan population [40].

We analysed equity in relation to condition incidence, service utilisation and expenditures across household wealth quintiles, measured by their reported monthly household consumption expenditure per adult equivalent. We estimated concentration indices to assess whether the distribution of outcomes was pro-rich (positive index value) or pro-poor (negative index value) [39] using the *conindex* command in Stata. The Gini index was used to estimate wealth inequality, and the Kakwani index, defined as the Gini index subtracted from the concentration index (CI), was used to estimate the progressivity of health expenditures [41]. The Gini index can vary between 0 and 1, with 0 indicating perfect wealth equality (the population having exactly the same wealth) and 1 maximum inequality. The Kakwani index can vary between -1 and 1, with negative values indicating the poor contribute a higher share of their income, or a regressive financing system, 0 indicating perfectly proportional expenditure and positive values indicating progressive contributions, whereby the richest pay a higher proportion of their income. Dominance tests were carried out to ascertain whether the concentration indices were significantly pro-rich or pro-poor, and whether the Kakwani index was significantly progressive or regressive.

We finally estimated the incidence of catastrophic and impoverishing health expenditure outcomes across the whole sample, and comparing refugee and host communities.

## Results

A total of 2533 women were interviewed in July-August 2019, of whom 1351 were refugees and 1182 were from host communities. The characteristics of the respondents and their households are given in Table 1, as well as comparison with national data drawn from the Ugandan National Demographic and Health Survey in 2016 [40]. Notably, 41.0% of refugee respondents were no longer married, or married but not living with a husband, and 71.4% were in female headed households, much higher proportions than seen in host communities (13.9% and 21.6% respectively) or nationally (13.5% and 31.0%). More refugee respondents (33.6%) lived in households of nine or more people, compared to only 19.1% in host communities, and 7.8% of households nationally. Education was poorer among refugees than hosts, but both compared unfavourably to the national average, with 31.0% of refugees having had no education, compared to 18.4% of host women and just 9.6% of women nationally. The mean annual consumption expenditure per adult equivalent was 653 USD (2011 PPP), or 1.79 USD per day, and similar across the two groups. However, the poorest three quintiles of refugees had higher mean consumption expenditure than the poorest three quintiles of hosts, while the wealthiest two quintiles had higher consumption expenditure among hosts.

**Table 1:** Respondent and household characteristics

	All respondents (*n*=2533)	Refugees (*n*=1351)	Hosts (*n*=1182)	P (chi2 test for difference between refugees and hosts)	2016 Demographic and Health Survey (Uganda)
	Frequency (percentage)				
Respondent age				0.014	% of women 15-49
15-19	862 (34.0)	456 (33.8)	406 (34.4)		23.0
20-29	1007 (39.8)	524 (38.8)	483 (40.9)		37.2
30-39	518 (20.5)	306 (22.7)	121 (17.9)		24.6
40-49	146 (5.8)	65 (4.8)	81 (6.9)		15.2
Respondent marital status				<0.0001	% of women 15-49
Never married	595 (23.5)	341 (25.2)	254 (21.5)		25.8
Married or living as married	1219 (48.1)	456 (33.8)	763 (64.6)		60.6
Married but not living with husband	486 (19.2)	422 (31.2)	64 (5.4)		13.5
Divorced, separated or widowed	233 (9.2)	132 (9.8)	101 (8.5)		
Respondent education				<0.0001	% of women 15-49
None	636 (25.1)	419 (31.0)	217 (18.4)		9.6
(Some) Primary	1512 (59.7)	713 (52.8)	799 (76.6)		57.4
(Some) Secondary or higher	385 (15.2)	219 (16.2)	166 (14.0)		32.9
Household head				<0.0001	% of households
Female	1219 (48.1)	964 (71.4)	256 (21.6)		31.0
Male	1266 (50.0)	351 (26.0)	915 (77.4)		69.0
Not specified	48 (1.9)	36 (2.7)	12 (1.0)		-
Household size				<0.0001	% of households
1-2	102 (4.0)	188 (1.3)	84 (7.1)		25.1
3-4	526 (20.8)	184 (14.4)	332 (28.1)		28.9
5-6	618 (24.4)	313 (23.2)	205 (25.8)		24.5
7-8	607 (24.0)	372 (27.5)	235 (19.9)		13.7
9+	680 (26.9)	454 (33.6)	226 (19.1)		7.8
Household annual consumption expenditure per adult equivalent	Mean consumption, USD 2011 PPP				
Overall	652.82	632.31	676.221	0.221	
1 (poorest)	165.93	189.16	145.58	<0.001	
2	350.72	387.98	312.67	<0.001	
3	530.46	557.66	492.81	<0.001	
4	759.68	750.05	780.00	<0.001	
5 (wealthiest)	1460.42	1287.82	1653.22	0.001	

A higher proportion of refugee women than host community women reported that a member of their household had been acutely sick in the two weeks preceding the interview (72.6 vs 65.2%) (Table 2). Incidence of acute sickness increased with wealth in both communities (Table 3), with 61.3% from the poorest households reporting a sick household member compared to 71.9% in the wealthiest households, and a positive (pro-rich) concentration index of 0.09 (*p*<0.01). Of those reporting acute sickness, wealthier families were also more likely to seek care across both communities, with 81.7% of the poorest households seeking care compared to 93.5% of the wealthiest, and a concentration index of 0.07 (*p*<0.01).

**Table 2:** Demand for and access to acute, chronic and maternal care for refugee and host community households

Outcome	Community	n/N	%	P (t-test for difference in means, refugees vs hosts)
Householdss with acute sickness in last two weeks	Total	1752/2533	69.2	<0.001
	Refugees	981/1351	72.6	
	Hosts	771/1182	65.2	
Sought care at a health facility (of households with acute sickness)	Total	1565/1752	89.3	0.095
	Refugees	887/981	90.4	
	Hosts	678/771	87.9	
Households with chronically sick member	Total	238/2533	9.4	0.691
	Refugees	130/1351	9.6	
	Hosts	108/1182	9.2	
Sought care at a health facility (of households with chronic sickness)	Total	180/238	75.6	0.107
	Refugees	93/130	71.5	
	Hosts	87/108	80.6	
Delivered a baby in Uganda in last year	Total	596/2533	23.5	<0.001
	Refugees	264/1351	19.5	
	Hosts	332/1182	28.1	
Had at least four ANC visits (of those who delivered a baby in Uganda in last year)	Total	407/596	68.3	0.899
	Refugees	181/264	68.8	
	Hosts	226/332	68.1	
Delivered in health facility (of those who delivered a baby in Uganda in last year)	Total	447/596	75	0.022
	Refugees	210/264	79.6	
	Hosts	237/332	71.4

**Table 3:** Demand for and access to acute, chronic and maternal care by wealth quintile

Outcome	Community	Q1 (poorest)	Q2	Q3	Q4	Q5 (wealthiest)	Concentration index
% of householdss with acute sickness in last two weeks	Total	61.3	67.3	73.8	71.3	71.9	0.09 (0.01) , *p*<0.0001
	Refugees	64.0	71.8	76.9	75.0	76.4	0.12 (0.01), *p*<0.0001
	Hosts	58.7	66.7	67.1	65.7	68.1	0.05 (0.02), *p*=0.0013
% sought care at a health facility (of households with acute sickness)	Total	81.7	91.0	89.6	90.2	93.5	0.07 (0.01), *p*<0.0001
	Refugees	85.1	92.5	87.5	90.3	95.7	0.08 (0.01), *p*<0.0001
	Hosts	77.7	88.8	91.1	89.7	91.3	0.05 (0.01), *p*<0.0001
% of households with chronically sick member	Total	6.9	9.3	8.7	9.1	13.5	0.11 (0.04) *p*=0.0030
	Refugees	7.7	11.1	6.6	8.0	14.6	0.12 (0.05) *p*=0.0139
	Hosts	8.5	5.8	9.4	10.6	11.5	0.10 (0.05), *p*=0.0758
% sought care at a health facility (of households with chronic sickness)	Total	65.7	79.2	70.0	75.0	83.6	0.05 (0.02) , *p*=0.0277
	Refugees	61.9	77.1	60.0	71.9	71.0	0.06 (0.04) *p*=0.1236
	Hosts	65.0	78.6	81.8	76.0	96.3	0.06 (0.03), *p*=0.0661
% delivered a baby in Uganda in last year	Total	24.5	22.6	27.2	20.9	21.7	-0.02 (0.02), *p*=0.2407
	Refugees	17.7	21.2	22.7	18.4	17.9	-0.01 (0.03), *p*=0.6647
	Hosts	31.2	24.2	29.5	29.2	26.4	-0.01 (0.03), *p*=0.6275
% had at least four ANC visits (of those who delivered a baby in Uganda in last year)	Total	68.6	80.2	69.9	63.7	56.1	-0.02 (0.01), *p*=0.0239
	Refugees	68.8	85.1	65.4	66.0	50.0	-0.03 (0.01) *p*=0.0156
	Hosts	70.3	70.7	72.5	66.7	59.7	-0.02 (0.01), *p*=0.1215
% delivered in health facility (of those who delivered a baby in Uganda in last year)	Total	80.7	68.1	73.7	71.6	81.6	0.00 (0.01), *p*=0.9963
	Refugees	83.3	70.2	78.9	79.3	90.9	0.02 (0.02), *p*=0.1863
	Hosts	75.7	70.7	78.3	60.9	71.0	-0.02 (0.02), *p*=0.2685

9.4% of women reported a chronically sick person in their household, with no difference between refugee and host respondents (Table 2). Wealthier households were more likely to report having a chronically sick member (Table 3), with 6.9% in the poorest quintile compared to 13.5% in the wealthiest quintile, and a concentration index of 0.11 (*p*<0.01). Among those reporting chronic sickness, similar proportions reported seeking care among host and refugee populations (75.6%), which were distributed broadly equally among wealth groups.

A higher share of host women (28.1%) compared to refugee women (19.5%) reported having delivered a baby in Uganda in the year preceding interview (Table 2), and this did not vary with wealth (Table 3). Levels of coverage of four or more ANC visits was similar among refugee and host populations at 68.3%. Among refugee women, access to ANC was better among poorer than richer women, with 68.8% of those from the poorest households having had at least four ANC visits, compared to only 50.0% from the wealthiest households (CI=-0.03; *p*<0.05). While poorer host women also had higher coverage than richer women, this difference was not statistically significant. Refugees had higher coverage of institutional deliveries than hosts (79.6% vs 71.4%, *p*=0.022), however in both samples the distribution was fairly even across wealth quintiles.

Total health expenditures were similar between hosts and refugees, at 74.87 USD and 73.91 USD respectively annual household healthcare expenditure (Table 4). However, hosts generally incurred higher expenditures for maternal care (mean 4.92 USD vs 2.87 USD, *p*=0.0093). In terms of the main cost drivers, expenditures associated with acute and chronic care seeking were the most substantial, with mean annualised expenditures of 100.47 USD and 138.04 USD respectively for those households who sought care. Only a small proportion incurred the chronic care costs due to the low incidence of chronic sickness, with only 180 households (7.1%) seeking care for chronic sickness. By comparison, expenditures for maternal care were very low, with mean annualised expenditure of 4.00 USD among those who sought care.

**Table 4:** Total, acute, chronic and maternal healthcare expenditure for refugee and host community households

Expenditure type	Community	N	Mean annualised household healthcare expenditure in USD	P (t-test for difference in means, refugees vs hosts)
Total healthcare expenditure (all households)	Total	2533	74.20	0.85
	Refugees	1351	73.61	
	Hosts	1182	74.87	
Acute healthcare expenditure (of those who sought care)	Total	1565	100.47	0.25
	Refugees	887	96.38	
	Hosts	678	105.81	
Chronic healthcare expenditure (of those who sought care)	Total	180	138.04	0.72
	Refugees	93	129.64	
	Hosts	87	147.02	
Maternal healthcare expenditure (of those who sought care)	Total	583	4.00	0.0093
	Refugees	262	2.87	
	Hosts	321	4.92	

In terms of the distribution of healthcare expenditures by wealth, wealthier households spent more on healthcare annually, with a mean annual expenditure of $146.96 in the highest quintile compared to $26.75 in the lowest (Table 5), and a significantly pro-rich concentration index of 0.297 (*p*<0.0001). However, the incidence of healthcare expenditure on households was still regressive, meaning that the poor paid a higher share of their total expenditure on health, as the high level of consumption expenditure inequality (Gini=0.409, *p*<0.0001) resulted in a negative Kakwani index (-0.112, *p*=0.00047). Greater wealth inequality (Gini=0.454 vs 0.366) and a lower concentration index (0.270 vs 0.333) among host households compared to refugees resulted in regressivity of healthcare payments among hosts but not refugees, with Kakwani indices of -0.186 (*p*=0.0002) and -0.033 (*p*=0.48) respectively. Expenditure on acute care was regressive in both communities, though more so among hosts, with a Kakwani index of -0.118 (*p*=0.0018) among refugees, and -0.260 (*p*<0.0001) among hosts. For spending on chronic care, expenditure was more concentrated in wealthier households, with a CI of 0.592 (*p*<0.0001) for refugees and 0.451 (*p*<0.0001) among hosts. The spending was progressive (but not statistically significant) for refugees (Kakwani index=0.233, *p*=0.260) and proportional for hosts (Kakwani=-0.004, *p*=0.977). For those who accessed maternal care (either ANC or delivery) in Uganda in the past year, there was higher expenditure by wealthier households among refugees (CI=0.151, *p*=0.015), but proportional expenditure within hosts (CI=0.087, *p*=0.197). Expenditure was regressive in both groups, but more so among hosts, with a Kakwani index of -0.361 (*p*<0.0001), compared to -0.199 (*p*=0.0025) in refugees. Overall, maternal care was the most regressive expenditure, followed by acute care, with chronic care costs being proportional.

**Table 5:** Total, acute, chronic and maternal healthcare expenditure for refugee and host community households

Expenditure type	Community	Mean annualised household healthcare expenditure by wealth quintile, USD					Concentration index	Gini index	Kakwani index (Concentration index – Gini index)
		Q1 (poorest)	Q2	Q3	Q4	Q5 (wealthiest)			
Total healthcare expenditure (all households)	Total	26.75	50.56	77.53	77.09	146.96	0.297 , *p*<0.0001	0.409, *p*<0.0001	-0.112 *p*=0.00047
	Refugees	27.23	49.13	74.47	70.15	159.57	0.333 *p*<0.0001	0.366, *p*<0.0001	-0.033, *p*=0.482
	Hosts	25.42	55.59	72.7	91.48	129.91	0.270 *p*<0.0001	0.456, *p*<0.0001	-0.186 *p*=0.0002
Acute healthcare expenditure (of those who sought care)	Total	47.98	74.10	104.52	108.39	158.43	0.208, *p*<0.0001	0.396, *p*<0.0001	-0.189 *p*<0.0001
	Refugees	47.87	67.01	104.15	95.89	164.42	0.243, *p*<0.0001	0.362, *p*<0.0001	-0.118, *p*=0.0018
	Hosts	47.30	84.82	104.85	130.35	147.10	0.179, *p*<0.0001	0.438, *p*<0.0001	-0.260, *p*<0.0001
Chronic healthcare expenditure (of those who sought care)	Total	25.78	39.61	92.84	81.97	329.31	0.511, *p*<0.0001	0.408, *p*<0.0001	0.102, *P*=0.402
	Refugees	19.44	34.87	61.19	67.46	339.45	0.592, *p*<0.0001	0.358, *p*<0.0001	0.233, *p*=0.260
	Hosts	26.67	36.81	69.65	145.62	308.39	0.451, *p*<0.0001	0.456, *p*<0.0001	-0.004, *p*=0.977
Maternal healthcare expenditure (of those who sought care)	Total	2.62	4.01	4.36	4.01	5.12	0.100, *p*=0.0556	0.405, *p*<0.0001	-0.305, *p*<0.0001
	Refugees	0.71	3.40	3.41	4.21	2.20	0.151, *p*=0.0152	0.350, *p*<0.0001	-0.199, *p*=0.00246
	Hosts	3.84	5.56	5.29	3.52	6.68	0.087, *p*=0.197	0.448, *p*<0.0001	-0.361, *p*<0.0001

In terms of financial protection, almost a third of households spent at least 10% of their annual expenditure on healthcare, and just under a fifth spent at least 25% (Table 6). Catastrophic expenditure was more common among host households with 19.8% spending at least 25% of total expenditure, compared to 16.4% among refugees, *p*=0.095. Catastrophic expenditure levels were higher for hosts than refugees for all the health conditions considered, though the difference was not statistically significant for chronic conditions (likely due to the small sample size). Acute and chronic care expenditures resulted in similar levels of catastrophic expenditure, whereas maternal care only resulted in catastrophic expenditures for a very small fraction of households. Poverty levels were similar between hosts and refugees prior to health expenditures, at 66.0% having per adult equivalent consumption expenditure below the international poverty line, but this was much higher than the Uganda-wide rate of 41.7% in 2016 [42]. Refugees were significantly more likely to be impoverished by health expenditure than hosts (17.2% vs 11.3%, *p*=0.015).

**Table 6:** Catastrophic and impoverishment effects of healthcare expenditure

	All respondents	Refugees	Hosts	P (chi square)
Catastrophic total healthcare expenditure at 10% of total expenditure	753/2533 (29.8%)	380/1351 (28.1%)	373/1182 (31.6%)	0.18
Catastrophic total healthcare expenditure at 25% of total expenditure	454/2533 (17.9%)	221/1351 (16.4%)	233/1182 (19.8%)	0.095
Catastrophic acute healthcare expenditure at 10% of total expenditure (of those who sought care)	675/1565 (43.1%)	354/887 (39.9%)	321/678 (47.4%)	0.055
Catastrophic acute healthcare expenditure at 25% of total expenditure (of those who sought care)	401/1565 (25.6%)	204/887 (23.0%)	197/678 (29.1%)	0.050
Catastrophic chronic healthcare expenditure at 10% of total expenditure (of those who sought care)	69/180 (38.3%)	32/93 (34.3%)	37/87 (42.5%)	0.26
Catastrophic chronic healthcare expenditure at 25% of total expenditure (of those who sought care)	47/180 (26.1%)	21/93 (22.6%)	26/87 (29.9%)	0.24
Catastrophic maternal healthcare expenditure at 10% of total expenditure (of those who sought care)	9/583 (1.5%)	1/262 (0.4%)	8/321 (2.5%)	0.048
Catastrophic maternal healthcare expenditure at 25% of total expenditure (of those who sought care)	2/583 (0.3%)	0/262 (0.0%)	2/321 (0.6%)	0.20
Poverty headcount before health expenditure	1671/2533 (66.0%)	886/1351 (65.6%)	785/1182 (66.4%)	0.659
Poverty headcount after health expenditure	1796/2533 (70.9%)	966/1351 (71.5%)	830/1182 (70.2%)	0.478
% Impoverished by health expenditure (of those not poor before health expenditure)	125/862 (14.5%)	80/465 (17.2%)	45/397 (11.3%)	0.015

## Discussion

We found a higher burden of acute sickness among refugees than hosts in this setting, though no difference in chronic care burden. This is a surprising finding, as while it is often suggested refugees have greater need for mental health and psychosocial support [43], there is little discussion of greater acute care needs [44]. The burden of both acute and chronic sickness increased with household wealth, which may be due to wealthier households being more likely to report these conditions [45], or due to greater health care seeking resulting in higher levels of diagnosis [46]. It could also be the case that chronic conditions such as diabetes are lifestyle related and more prevalent among wealthier quintiles [47]. Unmet need for acute and chronic care was more prevalent in poorer households among both hosts and refugees, but did not differ significantly between the two communities.

Patterns in need for and access to maternal care were variable. The need for maternal care (as measured by births in the last year) was higher among host communities than refugees. This may be as a result of births outside Uganda being excluded from the analysis, excluding some births to recent refugees. The lower birth rate may also be explained by refugee women being less likely to live with their husbands than host women. While ANC coverage was similar comparing refugee and host communities, poorer households generally had better access to ANC than wealthier ones. Micronutrient powders and fortified porridge given to refugees at ANC visits may be an incentive for poor women or those in food insecure households to attend antenatal clinics. Coverage of institutional deliveries was similar across wealth groups. Refugees were, however, significantly more likely than hosts to deliver at a health facility, and there are a number of potential explanations for this pattern. Many NGO workers and community health workers (often paid by NGOs) in refugee settlements provide health information and sensitise refugee women and communities about the importance of delivering in a facility, which may increase demand among these communities. There is anecdotal suggestion that health facilities in host communities may be less able to provide delivery care, with stockouts of delivery kits and lack of ambulances and trained health workers. Alternatively, it has been suggested that host areas are more likely to have skilled birth attendants who assist women delivering at home, disincenitvising them from coming to facilities. Finally, expense may be a factor, as extra costs such as transport to hospital are sometimes paid by humanitarian partners for refugees, but not for hosts. Previous research in Uganda also reported higher coverage of facility-based deliveries among refugees, but, in contrast to our study, reported higher coverage of ANC among hosts [17] .

Both host and refugee populations are poorer than the Ugandan national average. The mean annual consumption expenditure per adult equivalent in this sample was 653 USD (2011 PPP), or 1.79 USD per day. This is much lower than the mean annual consumption expenditure per adult equivalent from the Uganda National Household Survey of 2016/2017 which was 1.16 million Ugandan shillings [48], equivalent to 1085 USD in 2011 prices (PPP), or 2.97 USD per day. 66.0% of households had per adult equivalent consumption expenditure below the international poverty line of 1.90 USD (2011 PPP), much higher than the Uganda wide rate of 41.7% in 2016 [42].

Health expenditure was generally higher in wealthier households in this setting, but the high levels of consumption expenditure inequality meant than out-of-pocket expenditure was still regressive for most types of care. The one exception to this trend was chronic care, where expenditure was very highly concentrated among the wealthiest, and was somewhat progressive. The incidence of catastrophic expenditure was extremely high, and higher among hosts than refugees. This suggests that more needs to be done to improve the affordability of care for all living in these communities, and suggests that host communities should not be ignored while improving healthcare for refugees. Maternal care was very unlikely to result in catastrophic expenditure, suggesting that in this part of the health system, affordability has been well addressed, but care is needed to ensure the affordability of acute and chronic treatment. In contrast to findings on catastrophic expenditure, refugees were more likely than hosts to be impoverished by health spending, perhaps due to being more likely to be only just above the poverty line before health expenditure.

The finding that total out-of-pocket health expenditure was regressive among refugee and host communities is in contrast to other work in Uganda as a whole, which found that out-of pocket payments were slightly progressive [49]. This is likely to be because of difference between the refugees and hosts in this sample and the Uganda population. The same study conducted a benefit incidence analysis by facility level, finding a pro-rich distribution for hospitals (public and NGOs) and private clinics, but found that lower level public health units and drug shops were pro-poor. This also seems to be in contrast to our results, which, while they are not broken down by facility level, broadly find that healthcare utilisation is pro-rich, with the exception of ANC and delivery care.

Work looking at catastrophic and impoverishing health expenditure across Uganda in 2009/10 found that 22.8% of households experienced catastrophic health payments at the 10% level compared to 29.8% of households in our study, and 6.7% compared to 17.9% in our study at the 25% level [50]. This marked difference, particularly at the higher 25% threshold, suggests that both refugees and the communities that host them are more likely to experience catastrophic expenditure than the population generally. This could be due to both higher health needs and lower household incomes. The national analysis, using an earlier World Bank poverty line of $1.25, found a poverty headcount of 22.7% before healthcare expenditure and 26.8% after, with 5.3% of non-poor households being impoverished by healthcare expenditure. The 2015 change in poverty line to $1.90 means that a direct comparison with our figures of 66.0% and 70.9% living in poverty before and after healthcare expenditure is not appropriate, but the proportion impoverished is much smaller than our finding of 14.5% of non-poor households impoverished by health expenditure.

Our study highlights that while maternal care services are now largely affordable in Uganda among the refugee and host communities, and service access is generally pro-poor, the costs of acute and chronic care can be substantial, and access benefits wealthier groups, despite the abolition of user fees in public facilities [27]. The heavier reliance of households on public health services for maternal care [40] may partly explain these findings.

The random sampling approach and large sample size of this study gives confidence that the participants are representative of the population from which they are drawn, and that we can make accurate estimates of the use and costs of health services within these communities. However, there are some limitations in our methodology. For maternal health care, we only measured care received by women who have delivered a baby, not those who are currently pregnant or have miscarried, in order to be able to include ANC visits across the entire duration of pregnancy. However, if there is an association between receiving antenatal care and the pregnancy reaching full term, this may lead to overestimates of access to ANC. In terms of estimating expenditure, the survey was conducted with just one member of the household, who in about one third of cases was an adolescent under 20. It is possible that these respondents were less likely to be involved in the running of the household and have knowledge of the household finances, so responses from them may be less accurate. Wealth is estimated by asking what the household’s total expenditure was in the last month; this is a less accurate measure than asking for a breakdown of expenditure by category, and excludes consumption of food given by UNHCR or grown by households themselves. Healthcare expenditure does not take into account the opportunity cost and potential loss of income associated with seeking care.

The number of adults and children in the household is estimated: exact household size and make up was not available. In particular, any household with 9 or more members was recorded as “9+”; in our calculations we treated these households as having nine members, which is likely to have led to a consistent underestimation of household size (and overestimate of per capita and per adult equivalent wealth). Our method of estimating the number of adults in the household does not include any men, or women aged above 50, unless they were married to the respondent or were the head of the household. All other members of the household were assumed to be children. This may underestimate the number of adults and overestimate the number of children, also leading to an overestimate of per adult equivalent wealth. There were a substantial number of these large “9+” households in both refugee and host communities, but more among refugees (34% vs 19%), so while the poverty headcount is likely underestimated in both groups, this effect will be greater among refugees, and may bias the results towards a null hypothesis of no difference in poverty headcount between the groups.

## Conclusions

Our findings suggest that Uganda’s integration of host and refugee health services has been very successful in reducing differences in coverage between hosts and refugees for acute and chronic health care and ANC. However, more efforts are required to reduce differentials in delivery care access for hosts, and further research needed to unpack the reasons underpinning these differences. Greater investment in outreach and education services aimed at hosts, such as the existing Village Health Teams, could encourage more women to deliver at health facilities in host communities. Maternal care access is equitably distributed by wealth within host and refugee communities, and these services remain affordable. However, inequities in access within refugee and host communities remain for acute and chronic care, with efforts needed to enhance access among the poorest and reduce associated out-of-pocket payments and their impoverishing effects. Further research examining the cost drivers and potential financing arrangements to offset these will be important.

## Data Availability

The datasets used and/or analysed during the current study are not publically available to maintain participant confidentiality, but are available from the corresponding author on reasonable request.
